# Enhancer RNA: What we know and what we can achieve

**DOI:** 10.1111/cpr.13202

**Published:** 2022-02-16

**Authors:** Zhenzhen Han, Wei Li

**Affiliations:** ^1^ 117971 Stem Cell and Cancer Center The First Hospital of Jilin University Changchun China

## Abstract

Enhancers are important *cis*‐acting elements that can regulate gene transcription and cell fate alongside promoters. In fact, many human cancers and diseases are associated with the malfunction of enhancers. Recent studies have shown that enhancers can produce enhancer RNAs (eRNAs) by RNA polymerase II. In this review, we discuss eRNA production, characteristics, functions and mechanics. eRNAs can determine chromatin accessibility, histone modification and gene expression by constructing a ‘chromatin loop’, thereby bringing enhancers to their target gene. eRNA can also be involved in the phase separation with enhancers and other proteins. eRNAs are abundant, and importantly, tissue‐specific in tumours, various diseases and stem cells; thus, eRNAs can be a potential target for disease diagnosis and treatment. As eRNA is produced from the active transcription of enhancers and is involved in the regulation of cell fate, its manipulation will influence cell function, and therefore, it can be a new target for biological therapy.

## Graphical abstract

We introduce the production and characteristics of enhancer RNA (eRNA) as well as its functions to regulate gene expression and potential as a marker for the diagnosis and target for the treatment of cancer and other diseases.

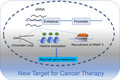

## 1 INTRODUCTION

Gene transcription is a very accurate regulatory process influenced by many factors, among which enhancers and promoters are important *cis*‐acting elements involved in the regulation of gene transcription. These *cis*‐acting elements can bind to *trans*‐acting factors that regulate transcription. Previous studies on *cis*‐acting elements have focussed on promoters; these elements initiate transcription in a position‐ and direction‐dependent manner. Meanwhile, enhancers are able to ignore these conditions as they can form a loop over long genomic ranges to approach distant promoters, thereby achieving specific gene expression.^1^, ^2^ Complexes, such as RNA polymerase II (RNAP II), transcription factors (TFs) and co‐regulators, can all bind to these *cis*‐elements. Although TFs can bind to both promoters and enhancers to form multi‐protein complexes that regulate transcription in a spatial and temporal fashion,^3^, ^4^ only promoters have the ability to initiate transcription by RNAP II. Recently, using high‐throughput sequencing technology, researchers have found that enhancers can be transcribed into RNA, referred to as eRNA.^5^

## 2 THE PRODUCTION OF ERNA

eRNA is mainly actively transcribed by enhancers that have the following features: (1) low levels of DNA methylation (low‐methylated regions)^6^, ^7^; (2) histone modifications at enhancer loci (eg H3K4me1 and H3K27ac)^8^, ^9^; (3) accessible (open) chromatin^10^; (4) TF occupancy, especially transcription initiation factors such as TBP, TFII and P300/CBP^11^; and (5) RNAP II occupancy.^7^

**FIGURE 1:**
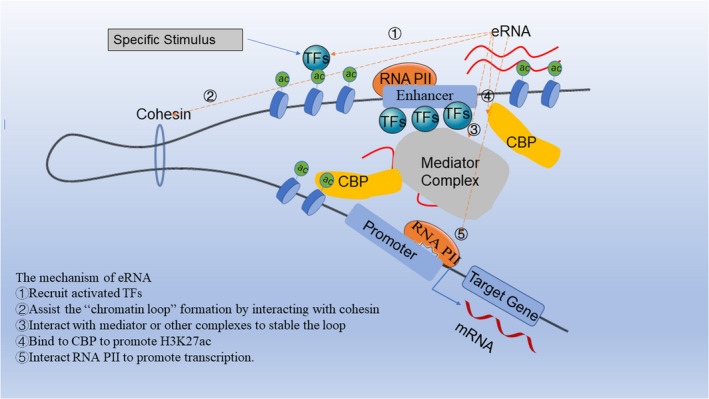
eRNA production and mechanism: First, activated TFs are recruited into the enhancer region to bind to specific DNA sequences and promote nucleosome remodeling under specific stimuli, allowing them to recruit other TFs, cofactors and complexes (eg mediator, p300/CBP). Second, CBP can acetylate H3K27 to promote accessible ‘open’ chromatin, thus recruiting RNAP II to promote eRNA transcription. Then, eRNA can interact with cohesion and other complexes to promote a ‘chromatin loop’, and it can promote the occupation of RNAP II in promoters to initiate the transcription of target genes

eRNA transcription generally occurs before the transcription of the code gene.^1^ Under specific stimuli, activated TFs are recruited into the enhancer region to bind to specific DNA sequences and promote nucleosome remodeling, which is a key factor affecting transcriptional activity.^12^, ^13^ Nucleosomes at enhancers possess a relatively high ratio of H3K4me1 to H3K4me3, and whereas the opposite is true for promoters, H3K4me2 occurs at both enhancers and promoters.^12^, ^14^ The active TFs recruit cofactors, other TFs and complexes (eg mediators and histone acetyltransferases) among which p300/CBP binds to many TFs through transactivation domains (TADs). The histone acetyltransferase (HAT) domain acetylates H3K27 to promote chromatin accessibility, thus recruiting bromodomain‐containing protein 4 (BRD4) and RNAP II.^15^, ^16^ More importantly, BRD4 can mediate the recruitment of transcription initiation cofactors forming the mediator complex and causing elongation of RNAP II in the hyperacetylated enhancer regions.^17^ Phosphorylation of the C‐terminal domain (CTD) of RNAPII is important for transcription and the stability of RNAP II.^18^, ^19^ Enhancers have high levels of the initiating form of RNAP II (Serine 5 phosphorylated, Ser5p), whereas the elongating form of RNAP II (Serine 2 phosphorylated, Ser2p) occurs infrequently at enhancers.^7^, ^20^ Ser5p can also be detected at promoters and directs bidirectional transcription, which is consistent with enhancer transcription.^21^, ^22^ The lower Ser2p occupancy at the enhancer may be caused by noncontinuous Pol II transcription, which contains a high density of poly(A) cleavage sites (PASs) that lead to early terminations. The transcriptional pausing and elongation of eRNAs are regulated by Spt5 and the positive transcription elongation factor b (P‐TEFb) complex. In contrast, eRNA termination can be regulated by a large complex named Integrator or the adaptor protein WD Repeat Domain (WDR82).^23^, ^24^ Previous publications show RNA modifications, such as m5C and m6A that also influence eRNA stability or metabolism, but whether this is a common phenomenon is unknown.^24^, ^25^, ^26^ Generally, the production of eRNAs involves a complex regulatory process and mechanism of action; however, they have been described in detail below. (Figure 1).

## 3 THE RELATIONSHIP BETWEEN ERNA, LNCRNA AND mRNA

The RNAs produced by enhancers and that produced by promoters are different, although they have many similarities. The mRNAs produced by promoters are long, spliced, polyadenylated and stable transcripts that can encode proteins in the cytoplasm. Long non‐coding RNAs (lncRNAs) are long and stable, usually spliced and polyadenylated, transcribed by their own promoters.^27^ Most eRNAs are short (about 500 bp), 5‐capped, non‐polyadenylated, unspliced and bidirectionally transcribed in the nucleus.^28^, ^29^ Only a small number of eRNAs are long (nearly 5 kb), polyadenylated and specifically transcribed from active enhancers.^20^ Polyadenylated eRNA is infrequent, and this may be due to degradation by nuclear RNA exosomes, which can degrade the 3′ end of eRNAs.^29^, ^30^ RNA exosome sensitivity inversely correlates to the distance between the transcriptional start site (TSS) and poly(A) signals (PAS). Thus, short eRNAs may have difficulty in the complete assembly of polyadenylation machinery on the CTD of RNAP II, leading to rapid degradation.^31^ This may explain why longer eRNAs are mostly polyadenylated and why most eRNAs are abundant, non‐polyadenylated and unstable. The longer, polyadenylated eRNA can perhaps be considered a class of lncRNAs but transcribed by enhancers instead of promoters.^15^ Super‐enhancers (SEs) are a group of clustered enhancers with broad Mediator and TF occupancy compared with that of typical enhancers.^32^ Recent reports show that eRNAs transcribed from SEs act as lncRNAs, while enhancer‐linked lncRNAs may function similarly to eRNAs; thus, the classification of eRNA and lncRNA is not entirely mutually exclusive.^33^, ^34^

## 4 THE INTERCHANGE BETWEEN ENHANCERS AND PROMOTERS

Enhancers were thought to regulate gene expression in an orientation‐independent manner, completely different from that of promoters. Interestingly, Mikhaylichenko et al. revealed that bidirectionally transcribed enhancers act as promoters in both directions.^35^ Using a transgenic assay with dual vectors proved that the promoter activity occurred predominantly in the same location with the elements that have endogenous enhancer activity, which means that enhancers and promoters both depend on the same regulatory components.^35^, ^36^ For enhancers to act as promoters depends on the level and directionality of eRNA transcription. Enhancers with high expression eRNA can generally function as promoters, and most bidirectional enhancers can act as weak promoters, producing overlapping spatiotemporal expression. In addition, some eRNAs are not short but extend into the downstream structural gene, and some intragenic enhancers can function as unidirectional, cell‐specific, alternative promoters to produce transcripts that share exons with their host genes but with low coding potential.^37^

Moreover, promoters can interact with enhancers and with other promoters to increase gene expression.^38^ Individual promoters are bidirectionally transcribed and have certain enhancer functions, so they can function as enhancers and promoters for the same gene.^39^ This is consistent with the fact that upstream promoter sequences and their upstream antisense TSSs can behave similarly to distal enhancers.^40^, ^41^ In fact, most promoters with unidirectional transcription cannot act as enhancers, and their endogenous transcriptional direction is associated with the direction when the element functioned as a promoter.

Research has shown that promoters and enhancers may have similar characteristics, such as sequence motifs, transcription machinery, chromatin environment, changes in activity upon binding of activators or repressors and H3K27ac occupancy.^40^, ^42^, ^43^, ^44^, ^45^ The shared features of promoters and enhancers indicate that there is no absolute difference between them, and they function depending on the context.^46^, ^47^ Furthermore, the balance between enhancer and promoter activity is generally reflected in the levels and directionality of eRNA transcription and is likely an inherent sequence property of the elements themselves.

## 5 FUNCTIONS OF ERNA

eRNA transcription has a strong correlation with enhancer activity, but the function of eRNA is controversial. Some researchers believe that eRNA is just a ‘noise’ product that is created when RNAP II complexes are recruited to the open chromatin environment of enhancers. Others have suggested that the complicated process of eRNA transcription is important for enhancer activation rather than the formation of eRNA itself. However, most researchers believe that the presence of eRNA itself acts as a transcription activator of the enhancer to promote the expression of the target gene.^10^, ^48^, ^49^

eRNA is produced by active enhancers; thus, they are responsible for tissue and lineage specificity and can be used to evaluate the cell type and state‐specific gene expression.^50^ eRNA transcription can be initiated by specific stimuli, and they are dynamically regulated by signalling molecules such as NFκB and p53 in different conditions.^51^, ^52^ eRNA can be a symbol of enhancer activity, and the regulation of eRNA can be influenced by mRNA levels at proximal genes,^10^, ^53^, ^54^ whereas eRNA can influence its target genes. Generally, the level of eRNA is positively correlated with the target mRNA, and the knockdown of eRNA leads to decreased target‐gene expression.^55^, ^56^

SERPINB2, a strongly expressed gene in inflammatory states, can be upregulated by upstream eRNA, which is transcribed prior to SERPINB2 mRNA. Knockdown of eRNAs inhibited the transcription of SERPINB2 mRNAs, while eRNA overexpression was associated with upregulated SERPINB2 mRNA. This provides direct evidence that control of eRNA expression can influence mRNA levels.^57^

In macrophages, Rev‐Erbs nuclear receptors regulate the expression of Mmp9 and Cx3cr1 by reducing the transcription of distal enhancers selected by macrophage lineage determinants.^58^ Furthermore, Rev‐Erbs can directly mediate the targeted degradation of eRNAs, thus leading to decreased expression of nearby genes. When the Rev‐Erb factors are decreased, the relative enhancers can become active, resulting in high transcription of Mmp9 and Cx3cr1 mRNAs.^59^, ^60^ This provides evidence that eRNAs can function in the selective regulation of target genes. Another study showed that enhancers influence promoters depending on the sequences mediating TF binding and the sequences encoding the eRNA transcript.^58^

The transcription of eRNA is closely associated with the regulation of nearby target genes, and knockdown of eRNA decreases the expression of relative target genes.^5^, ^61^, ^62^ In this context, reduced eRNA transcription by RNA interference (RNAi) or antisense oligonucleotides (ASO) leads to the reduction of nascent protein‐coding genes. Other studies have shown that exogenous overexpression of actual eRNA sequences can promote an increase in respective mRNAs.^12^, ^51^, ^54^, ^58^ More importantly, when the orientation of the coding sequence region was plus strand and reversed relative with the minus ‘core’ enhancer RNA, the plus‐strand eRNA sequences did not provide a potentiating effect. For example, Cx3cr1 has a 28kb minus‐strand eRNA expression, and the minus‐strand eRNA restored the activity of the core enhancers, but the plus strand did not. Besides, Mmp9 expresses a −5kb plus‐strand eRNA, and the overexpression of the plus‐strand eRNA, but not that of minus‐strand eRNA, restores the transcriptional activity when siRNAs or ASOs directed against the plus‐strand eRNA result in a reduction of Mmp9 mRNA expression.^58^ The above description explains the importance of the specific eRNA sequence in promoting transcription compared ‘missense’ RNA transcripts.

## 6 MECHANISM OF ACTION

As the majority of eRNAs are non‐polyadenylated, short‐lived and unstable, they act in *cis*, ie intrachromosomally.^29^ Meanwhile, the other long, stable, poly‐A eRNAs can affect the expression of abundant genes by relocating to other chromosomal regions—not the region in which they are transcribed—therefore, they act in *trans*, ie interchromosomally.^63^, ^64^ For example, the eRNA transcribed from a myogenic differentiation 1 (MYOD) distal regulatory enhancer (DRR), also called DRR eRNA, can regulate the expression of other chromosomal target genes in *trans*.^63^ KLK3 eRNAs can also enhance androgen receptor‐dependent gene expression in *trans* in human prostate cancer.^65^

### 6.1 Promoting the formation of the ‘chromatin loop’

Enhancers can span the space to regulate the expression of one or more genes, forming the ‘chromatin loop’ structure to take the enhancer to the vicinity of the target‐gene promoters, thereby fully approaching and interacting with the target gene in space by activating or inhibiting the target gene. TFs, transcriptional coactivators, steroid hormone receptor, CTCF, cohesion and other protein complexes play an important role in the construction of the ‘chromatin loop’.

TFs can bind to the specific recognition sequences of enhancers, even when the enhancer is located far from where the transcription initiation complex is bound.^66^ The TFs that bind to enhancers can also bind co‐activators, such as the Mediator and p300, to the transcription initiation complex bound to the promoter to regulate gene expression.^67^, ^68^ Mediators are transcriptional co‐activators that contain subunits such as Med6, Med7, Med10, Med12, Med14, Med15, Med17, Med21, Med24, Med27, Med28 and Med30^69^; they promote the interaction between TFs on the enhancers and the transcription initiation complex, especially RNAP II on promoters.^70^ The Mediator also promotes RNAP II occupancy and the formation of transcription initiation complexes at the promoter.^71^

Cohesin has been proved to occupy sites bound by CTCF and participate in the formation of the ‘chromatin loop’ by connecting two DNA segments.^72^, ^73^ It is a complex containing Smc1a, Smc3, RAD21 and Stag2, with a loading factor Nipbl that provides a way to load cohesin at promoters. Recent research suggests that cohesin can also occupy the enhancer and core promoter sites bound by the Mediator, and the co‐occupied region is associated with RNAP II.^74^ The ‘chromatin loop’ between enhancers and core promoters can be formed by the interaction between enhancer‐bound TFs, mediators, cohesin and promoter‐bound RNAP II.

Enhancers involved in the ‘chromatin loop’ with core promoters have high expression of eRNA, suggesting that eRNAs may act in the formation of the ‘chromatin loop’.^50^ Estrogen receptor α (ERα) is a TF that can interact with cohesin subunits, leading the enhancer to the target‐gene promoters to form this ‘chromatin loop’. β‐estradiol (E2)‐bound ERα on enhancers also causes a global increase in eRNA transcription.^54^, ^75^ Using siRNA to reduce eRNAs results in a decrease in cohesin recruitment to enhancers and reduces the interaction of enhancers and promoters, finally suppressing the expression of target genes. RNA pull‐down and RIP‐QPCR show that eRNAs can interact with SMC3 and Rad21 subunits of the cohesin complex, which is important in the formation of ‘chromatin loop’. SiRNA‐mediated depletion of SMC3 and Rad21 also influences the loop and expression of related genes.^54^ To summarize, eRNA can promote target‐gene expression by interacting with SMC3 and Rad21 subunits of cohesin complex to form a ‘chromatin loop’.

### 6.2 Regulation of histone modification

An active enhancer has an accessible chromatin architecture accompanied by enhanced DNase I hypersensitivity.^76^ In contrast to other free RNA, eRNA can interact with chromatin to modify chromatin accessibility and impact various histone marks, especially the acetylation and methylation of H3K27.^57^, ^63^, ^76^ Histone acetylation and CBP/p300 co‐activator binding is a common feature of active enhancers. eRNAs can directly interact with CBP/P300 to stimulate histone acetyltransferase activity, and increased CBP/p300 activity promotes more H3K27ac at the enhancer and the target promoter.^16^ The knockdown of eRNA reduces H3K27ac levels on enhancers and target‐gene promoters.^55^, ^56^ eRNA displaced the activation loop from the catalytic site and increased the affinity of CBP for its histone substrate. Earlier, we had mentioned that active enhancers have a high ratio of H3K4me1 to H3K4me3, and eRNA can decrease the level of repressive H3K4me3.^56^ This may be caused by the interaction between eRNA and polycomb repressive complex 2 (PRC2). PRC2, composed of the EZH2 and SUZ12 subunits, represses transcription by increasing H3K27me3 levels.^77^ The nascent RNA, including eRNA, binds to the EZH2 subunit to antagonize its nucleosome‐binding activity and represses the methyltransferase activity of PRC2, thereby reducing H3K27me3 to promote gene transcription.^78^, ^79^ A typical example is that of eRNA CARMEN, which was shown to interact with EZH2 and SUZ12 to change the cardiac precursor cell's fate through epigenetic regulation.^80^ Generally, eRNA can not only activate acetyltransferases to increase the H3K27ac levels but also reduce the repressive H3K4me3 levels, and eRNA production is regulated by demethylases like Kdm6a/b.^81^ Therefore, we can conclude that eRNAs promote a feed‐forward loop of eRNA production and histone modification associated enhancer activation.

### 6.3 Recruitment of RNAP II

In accordance with the central role of RNAP II in the ‘chromatin loop’‐mediated transcription,^82^ we propose that eRNA can recruit RNAP II to promote transcription initiation complex loading and assembly. MYOD and MYOG can bind some extragenic locations with active enhancer signature (ie high H3K4me1‐to‐H3K4me3 ratio, acetylated histones, and Pol II‐occupied) and generate RNA. In the regulation of MYOD1, core enhancers can be transcribed into CERNA, which elevates the RNAP II load on the target‐gene promoters.^52^ Reducing the transcription of CERNA leads to a decrease in Pol II abundance at promoters and gene bodies of MYOD1 and MYOG loci and influences chromatin accessibility (can be measured by Dnase‐seq), which is a rate‐limiting step preceding RNAP II assembly, but not on the core enhancer itself.^83^

In addition, eRNAs promote the transition from paused RNAP II into active elongation by acting as decoys for the negative elongation factor (NELF) complex upon induction of immediate early genes (IEGs) in neurons. Knockdown of eRNAs impairs the transient release of NELF from the target promoters during transcription, resulting in a reduction in the elongating form of RNAP II, thus leading to a decrease in target mRNA expression.^84^

## 7 ERNA AND TUMOURS

Using the Cancer Genome Atlas (TCGA) RNA‐seq database, we found higher concentrations of eRNAs in a broad range of cancers relative to those in normal tissues.^85^ Integrative analysis with multi‐omics data from TCGA, CCLE, ENCODE, FANTOM, Roadmap Epigenomics and pharmacogenomics datasets from GDSC and CTRP provided information on the role of eRNAs in the development of different cancer and eRNA‐targeted tumour treatments.^86^, ^87^ Moreover, further experiments have proved that eRNA may be involved in the regulation of oncogenes, tumour‐suppressor genes, and abnormal cancer signalling pathways of external signals.^88^

### 7.1 KLK3e in prostate cancer

Androgen receptors (ARs) can influence the development of prostate cancer by binding at different gene loci to initiate transcripts, leading to important cellular activities.^89^

**FIGURE 2:**
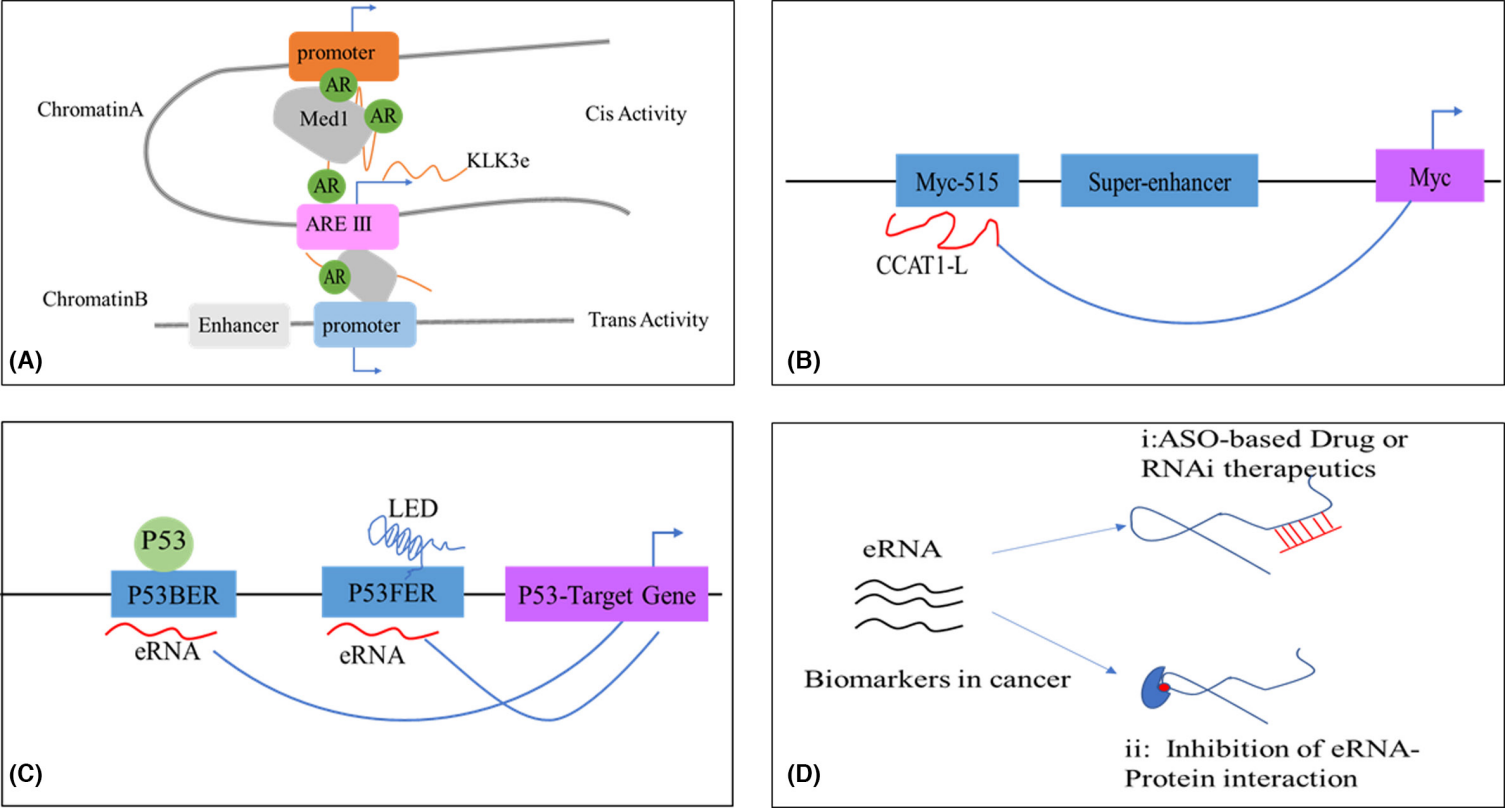
eRNA and tumours. eRNAs are numerous in tumour cells but can also be specific to specific tumours. Here, we summarize the tumour eRNA mentioned in this review. (A) In prostate cancer, Klk3e is transcribed from androgen response element III (ARE III), and some KLk3e promote gene transcription in *cis*, ie intrachromosomally (KLK3/KLK2). However, some eRNAs can affect the expression of abundant genes by relocating to chromosomal regions distinct from those in which they are produced, in *trans*, ie interchromosomally. (B) CCAT‐L is an eRNA transcribed from a locus 515 kb upstream near an SE of MYC, and can regulate MYC expression by promoting the formation of long‐range chromatin looping. (C) p53 can bind to p53‐bound enhancer regions (p53BERs) to produce eRNAs, while genes that are p53‐free enhancer regions (p53FERs) can be activated by LED to produce eRNAs. (D) eRNAs can be a biomarker for cancer therapy through RNAi‐based therapy or inhibitors to suppress the eRNA–protein interactions

Kallikrein‐related peptidase 3 (KLK3), a gene encoding prostate‐specific antigen (PSA), can be regulated by AR, and its enhancer is known as androgen response element III (ARE III) marked by AR, H3K27ac and H3K4me1.^90^ Thus, the KLK3 enhancer may be transcriptionally active according to the description above, and this region produces an eRNA named KLK3 eRNA (KLK3e), as confirmed by GRO‐seq data and RT‐qPCR analyses in androgen‐dependent LNCaP and VCaP, and androgen‐independent LNCaP‐abl cells.^65^ KLK3e can be bidirectionally transcribed, but sense strand KLK3e plays a role in the AR‐induced regulatory network. KLK3e promotes the interaction of KLK3 enhancer and KLK2 promoter, forming a loop complex with AR and Mediator1 (Med1) to facilitate the transcription of KLK2. The expression of KLK3e is correlated with KLK3 and KLK2 in prostate cancer. By using siRNA to silence KLK3e, we found that not only KLK2 and KLK3 were influenced but also the expression of NKX3.1, FKBP5 and PLZF beyond the KLK locus was decreased. This proved that eRNA can lead an AR‐associated protein complex to target chromatin and determine the specific transcription either intrachromosomally (*cis* activity) or interchromosomally (*trans* activity) (Figure 2A). Moreover, reduced expression of KLK3e suppresses the growth of prostate cells.^65^ Besides KLK3e, we verified that there are many other AR‐regulated enhancer RNAs (AReRNAs) that are increased in castration‐resistant prostate cancer (CPRC) cells.^91^ This suggests that reducing AR‐eRNAs by ASOs can be a new target for CRPC therapy.^92^

### 7.2 The influence of CCAT1 on tumour growth

MYC is an important proto‐oncogene that influences tumour development in many tumour types.^93^ CpG island methylator phenotype (CIMP) colorectal cancer has poor patient outcomes and depends on bromodomain and extraterminal (BET) activity for c‐MYC transcription. McCleland et al. identified a non‐coding RNA transcribed from the c‐MYC enhancer, called CCAT1 (colon cancer‐associated transcript 1), through integrated transcriptomics and ChIP‐seq.^94^ CCAT1 is highly associated with c‐MYC expression and cell growth in most cancers. CCAT1‐L, a longer form of CCAT1, specifically transcribed from a locus 515 kb upstream near a SE of MYC, can regulate MYC expression by promoting the formation of long‐range chromatin looping^95^(Figure 2B). Overexpression of CCAT1L increases the expression of MYC and tumour growth, while knockdown of CCAT1 reduces the expression of MYC and inhibits tumour growth.^94^, ^95^ CCAT1 is a BET transcriptional target and marker for cells dependent on BET‐mediated c‐MYC transcription; thus, it can be a clinical marker to recognize which patients may benefit from BET inhibitors. This provides direct evidence that eRNAs can regulate the expression of proto‐oncogenes to influence tumour growth.

### 7.3 Ernas and p53

P53 is a key tumour‐suppressor gene that can bind to the enhancer region of target genes to determine cell fate.^96^ The p53‐bound enhancer regions (p53BERs) are active and have high levels of H3K27ac and H3K4me1, and p53BER can produce eRNA to regulate selective gene expression in a p53‐dependent manner.^51^ We reduced the expression of eRNA produced from p53BER by siRNA, by inhibiting gene transcription close to the p53BER domains such as PAPPA and IER5 when p53 is activated by nutlin‐3. However, not all eRNA‐producing enhancers have p53BER, but they can be activated by p53‐induced lncRNAs, among which the lncRNA activator of enhancer domains (LED) can interact and activate the enhancer to produce eRNA.^97^ LED is be located near the enhancer of the CDKN1A gene, which is a potent p53‐responsive cell‐cycle inhibitor. Knockdown of LED suppresses the CDKN1A enhancer activity of tumour cells following p53 activation, and in most human tumours, LED is silenced to suppress p53 activation.^97^ Therefore, as a co‐factor with p53, LED can regulate the transcription of eRNA in a nutlin‐3a‐dependent manner. (Figure 2C).

Based on the cancer‐specific expression of eRNAs, we suggest that eRNAs are valid diagnostic or prognostic biomarkers in cancer therapy. Moreover, some eRNAs can be utilized to determine cancer‐related clinical features, such as subtype, targets for cancer therapy, stages and grades.^86^ Oncogene expression can be regulated by using ASO or RNAi‐based therapies or by suppressing the eRNA–protein interaction (Figure 2D).

## 8 ERNA AND STEM CELLS

Embryonic stem cells (ESCs) have the properties of self‐renewal and pluripotency, and their transcription can be affected by specific TFs such as Oct4, Sox2, Nanog and Sall4.^98^, ^99^, ^100^, ^101^ Methylation and acetylation are important epigenetic markers that define the signatures of ESCs. eRNA‐producing enhancers are active in ESCs and are called ‘super enhancers’; they promote these properties of self‐renewal and pluripotency.^7^ Research has shown that lncRNA *Peblr20* binds to the Oct4 enhancers and recruits the TET2 demethylase to promote enhancer transcription, thereby controlling the fate of stem cells.^102^ There is an enhancer element 45kb upstream of the Nanog locus, labelled with H3K4me1 and H3K27ac, and it is occupied by Nanog, Oct4, Sox2, Med1 and Med12, and p300 also binds to it.^103^ In contrast, p300, Med1 and Med12 binding was not observed in MEF cells. Nanog‐linked eRNA is produced in an ESC‐specific fashion, regulated not only by pluripotent factors but also by Tet1 and Tet2, which are predominant in ESCs.^104^ During differentiation, the Nanog enhancer can be silenced by histone demethylase lysine‐specific demethylase 1 (LSD1) which can demethylate histone H3 on Lys 4 or Lys 9 (H3K4/K9), thereby decreasing Nanog‐linked eRNA and mRNA.^7^, ^105^ In ESCs, LSD1 can interact with Oct4‐directed enhancers but cannot demethylate histone H3K4, with similar occurrences in the presence of acetylated histones of p300.^106^ However, p300 decreases with differentiation, leading to a decrease in acetylated histones, which leads to demethylation of H3K4 by LSD, thus suppressing the enhancer from producing eRNAs.

## 9 ERNA AND PHASE SEPARATION

We have mentioned that SEs bind with many TFs to control gene transcription.^32^, ^107^ Recent research has shown that they exist as liquid‐liquid phase‐separated biomolecular condensates.^108^ The phase‐separated structures of SEs are composed of scaffolding nucleic acids and proteins with intrinsically disordered regions (IDRs). IDR is a protein feature that facilitates condensate formation,^109^, ^110^ and it is involved in the construction of the coactivators BRD4 and MED1 at sites of SE‐driven transcription.^108^ RNA is a scaffold that plays a direct role in the formation of phase‐separated structures, among which lncRNA NEAT1 is a scaffold of paraspeckles.^111^ A recent study showed that MegaTrans enhancers produce eRNA to construct enhancer ribonucleoprotein (eRNP) complexes with condensins to exhibit properties of phase‐separated condensates.^112^ The eRNA is the scaffold to enhancer phase separation, and the respective enhancers are called ‘phase‐separated enhancers’ (PSE).^15^ Thus, PSEs are rich in IDR proteins, and eRNA can induce phase separation by interacting with the RBD in the IDRs of these proteins.

## 10 CURRENT LIMITATIONS AND PROPOSAL FOR FUTURE STUDIES

Although the existence of eRNAs is not debatable, there are still many questions about their mechanism and specific applications. The secondary structure of eRNA is unknown, and it is also unknown how the structure influences cell function. Proteomic methods such as ChIRP, RAP and iDRiP can be used to recognize tumour‐specific eRNA‐protein interactions, and this can be further used to explore what role they play in the progression of tumours.^113^, ^114^, ^115^ Another interesting research area is the exploration of the occurrence of RNA modifications such as methylation by N6‐adenosine (m6A) in eRNAs and whether these modifications play specific roles in cells. Moreover, eRNA is involved in phase‐separated condensates, and further research may help to address the relationship between eRNA production and phase separation.^116^

## 11 CONCLUSION

Enhancers and promoters have many similar features. eRNA is produced from enhancers and is involved in the selective expression of specific genes. Most eRNAs are short, 5‐capped, non‐polyadenylated, and only a small portion of eRNAs are long and polyadenylated. As most eRNAs are not stable, global run‐on sequencing (GRO‐seq), precision nuclear run‐on sequencing (PRO‐seq), chromatin immunoprecipitation (ChIP)‐seq or cap analysis of gene expression (CAGE) sequencing is used to recognize eRNAs instead of the common RNA‐seq.^53^, ^117^, ^118^, ^119^ eRNAs can regulate target genes by changing the chromatin spatial conformation, promoting enhancer‐promoter interactions and gene transcription.

An increasing number of studies have shown that eRNAs are involved in tumour oncogenic signalling pathways and manipulation of tumour genesis. Most eRNAs have specific expression patterns with associated TFs, and they are related to cancer types. KLK3e, NETIe and CCAT1 are eRNAs that can be found in specific tumours, and their suppression will influence tumour progression. eRNA can be used for cancer prevention, early diagnosis and therapy. Many studies have shown that most disease‐associated loci represent sequence variations in enhancers.^120^, ^121^ A database of eRNAs in cancer (eRic) can explore eRNA's role in cancer.^86^ Moreover, eRNAs can be involved in the maintenance of stem‐cell self‐renewal and pluripotency.

Previous studies have shown that many disease‐associated genetic variants occur in putative enhancer elements.^122^ If the limitation described above can be addressed, we may be able to obtain a thorough understanding of eRNAs that can enable us to manipulate them in tumours and stem cells.

## CONFLICT OF INTEREST

The authors declare no conflict of interest.

## AUTHOR CONTRIBUTIONS

All authors contributed to the review conception and design. Zhenzhen Han wrote the manuscript; Wei Li revised and approved the manuscript.

## Data Availability

Data sharing is not applicable as no new data were created or analyzed in this study.
